# A new target of multiple lysine methylation in bacteria

**DOI:** 10.1128/jb.00325-24

**Published:** 2024-12-11

**Authors:** Shori Inoue, Shogo Yoshimoto, Katsutoshi Hori

**Affiliations:** 1 Department of Biomolecular Engineering, Graduate School of Engineering, Nagoya University 542239 , Nagoya, Aichi, Japan; CBI, NLM, National Institutes of Health, Bethesda, Maryland, USA

**Keywords:** posttranslational modification, protein lysine methylation, trimeric autotransporter adhesin, bacterial adhesion

## Abstract

The methylation of ε-amino groups in protein lysine residues is an important posttranslational modification in eukaryotes. This modification plays a pivotal role in the regulation of diverse biological processes, including epigenetics, transcriptional control, and cellular signaling. Recent research has begun to reveal the potential role of methylation in modulating bacterial immune evasion and adherence to host cells. In this study, we analyzed the cell surface proteins of the toluene-degrading bacterium *Acinetobacter* sp. Tol 5 by label-free liquid chromatography‒mass spectrometry and found multiple lysine methylation in its trimeric autotransporter adhesin (TAA), AtaA. Over 130 lysine residues of AtaA, consisting of 3,630 amino acids and containing 234 lysine residues, were methylated. We identified that the outer membrane protein lysine methyltransferase (OM PKMT) of Tol 5, KmtA, specifically methylates the lysine residues of AtaA. In the KmtA-deficient mutant, most lysine methylations on AtaA were absent, indicating that KmtA is responsible for the methylation of multiple lysine residues throughout AtaA. Bioinformatic analysis revealed that the OM PKMT genes were widely distributed among Gram-negative bacteria, including pathogens with TAAs that promote infectivity, such as *Burkholderia mallei* and *Haemophilus influenzae*. Although KmtA has sequence similarities to the OM PKMTs of *Rickettsia* involved in infectivity, KmtA-like PKMTs formed a distinct cluster from those of the *Rickettsia* type according to the clustering analysis, suggesting that they are new types of OM PKMTs. Furthermore, the deletion of Tol 5 KmtA led to an increase in AtaA on the cell surface and enhanced bacterial adhesion, resulting in slower growth.

**IMPORTANCE:**

Lysine methylation has been underexplored in prokaryotes, and information on it is limited to some pathogens. Our finding is the second case of multiple lysine methylation of bacterial outer membrane (OM) proteins, following that of OmpB of *Rickettsia*. The newly found target of methylation, AtaA, a trimeric autotransporter adhesin family protein of *Acinetobacter* sp. Tol 5 isolated from activated sludge, extends our understanding of OM protein methylation to non-pathogenic environmental strains. The newly identified enzyme KmtA shows higher specificity than rickettsial lysin methylases, protein lysine methyltransferases, and methylates more lysine residues of the target, which raises interest in the mechanism underlying its biological specificity. The widespread presence of KmtA-like PKMTs throughout Gram-negative bacteria suggests that lysine methylation functions more extensively in bacterial physiology than previously recognized.

## INTRODUCTION

Bacterial fiber proteins on the cell surface are essential mediators of numerous biological functions, including cell adhesion, biofilm formation, motility, DNA transfer, and interaction with host cells (1–4). These proteins are critical for bacterial survival and facilitate infection and contamination by pathogenic bacteria (5). On the other hand, fiber proteins from beneficial bacteria have been used for developing various technologies, such as cell-surface display, cell immobilization, bioremediation, and self-repairing materials (6–8).

The intricate structure and composition of these fiber proteins enable a variety of biological functions. Flagella and pili are the major fiber proteins present in a wide range of bacteria. These proteins are composed of thousands of subunits, and their assembly is governed by complex, highly regulated genetic and biochemical processes that ensure their structural integrity and functions (9–11). In addition, many Gram-negative bacteria have autotransporter adhesins, which are secreted by the type V secretion system and consist of a passenger domain that is displayed on the outer membrane (OM) and a transmembrane domain that functions as a translocator (12).

The toluene-degrading bacterium *Acinetobacter* sp. Tol 5 exhibits autoagglutination and extremely high adhesiveness to various types of solid surfaces and has been studied for application as an immobilized whole-cell catalyst (13, 14). The high adhesiveness of Tol 5 is mediated by its fibrous cell surface protein AtaA, which is a member of the trimeric autotransporter adhesin (TAA) family, a subtype of autotransporters. The polypeptide chains of AtaA, which consists of 3,630 amino acids, form a very large homotrimeric fibrous structure that is 260 nm in length (14). Recent studies have revealed that Tol 5 cells exhibited strong adhesion force to almost all types of materials, but not to highly hydrophilic 2-methacryloyloxyethyl phosphorylcholine polymer surfaces (15). The molecular structure and functional domains of AtaA were also investigated (7, 16). On the other hand, electron microscopy has revealed the presence of various fiber proteins other than AtaA on the cell surface of Tol 5 (17). However, a comprehensive analysis of these fiber proteins on the cell surface of Tol 5 has yet to be conducted.

In this study, we first analyzed the proteome of Tol 5 cells using liquid chromatography‒mass spectrometry (LC‒MS) and successfully identified a variety of fiber proteins. LC‒MS analysis coincidentally revealed that the numerous lysine residues of AtaA, the most abundant fiber protein on the cell surface of Tol 5, were methylated. Protein lysine methylation is a common posttranslational modification, in which the ε-amino group of a lysine residue can be mono-, di-, or trimethylated by protein lysine methyltransferases (PKMTs) (18). While protein lysine methylation has been studied mainly in histones and various nonhistone proteins of eukaryotic cells (19–21), recent studies have revealed that multiple lysine residues on some cell surface proteins are methylated in prokaryotes, such as *Rickettsia* and *Salmonella* species, playing crucial roles in the host immune response during infection and adhesion to host cells (22, 23). However, to our knowledge, there are no other reports of multiple lysine methylation in bacteria. Therefore, in this study, we focused on the multiple lysine methylation of AtaA, identified the enzyme responsible for the methylation, and examined the effects of the methylation on the biogenesis of AtaA and the physiology of Tol 5.

## RESULTS

### Identification of fiber proteins on the Tol 5 cell surface

**Fig 1:**
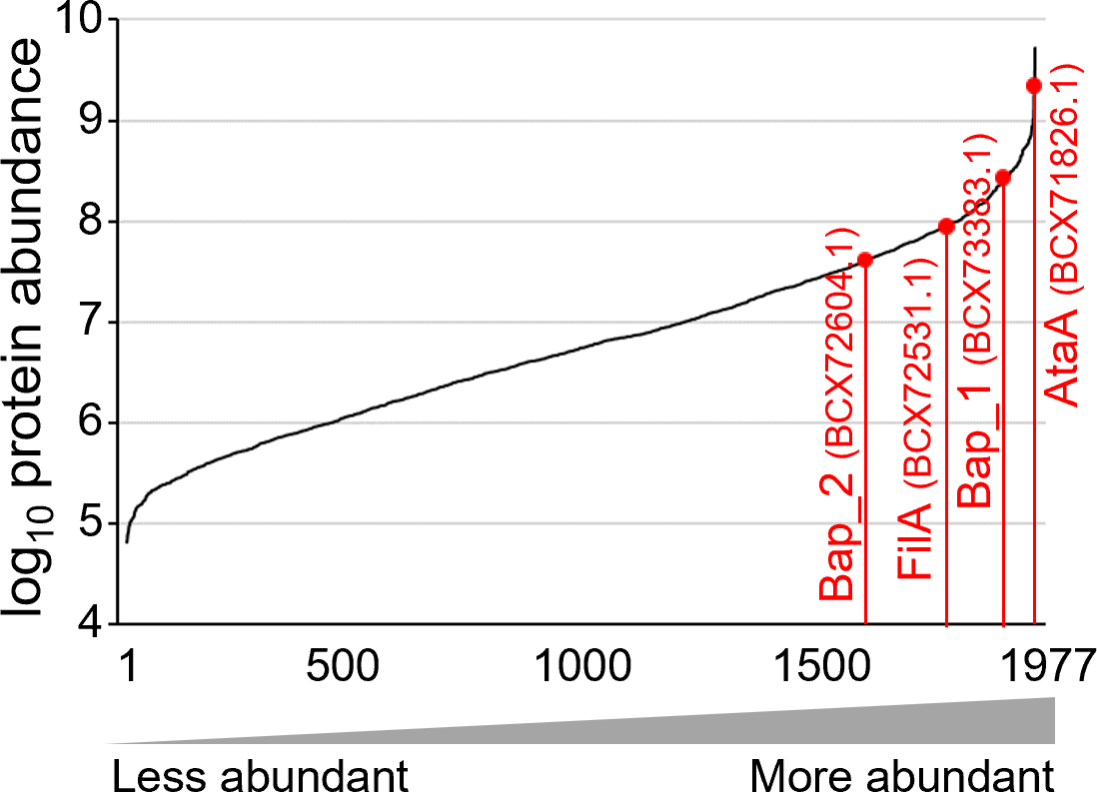
Ranked protein abundance in Tol 5 cells. The abundances of all quantified proteins were plotted based on quantitative label-free proteomics.

We performed label-free LC‒MS analysis on trypsin-digested peptides from Tol 5 cells and detected 1,977 proteins. These proteins included previously reported cell surface fiber proteins such as AtaA (BCX71826.1) and the Fil pilus subunit FilA (17) (BCX72531.1) (Fig. 1; Table S1). In addition, we detected the biofilm-associated proteins (Baps) (BCX73383.1 and BCX72604.1), which are secreted onto the cell surface by the type I secretion system and are involved in biofilm formation in *Acinetobacter baumannii* (24). The chaperone and usher of the F17-like pilus (BCX73228.1 and BCX73229.1) were also detected, but its main subunit (BCX73227.1) was not detected. In *A. baumannii,* the Csu pilus was reported to facilitate abiotic surface adherence and biofilm maturation (25), but proteins with sequence similarity to those of the Csu pilus (BCX73208.1–BCX73213.1) were not detected in Tol 5.

**Fig 2:**
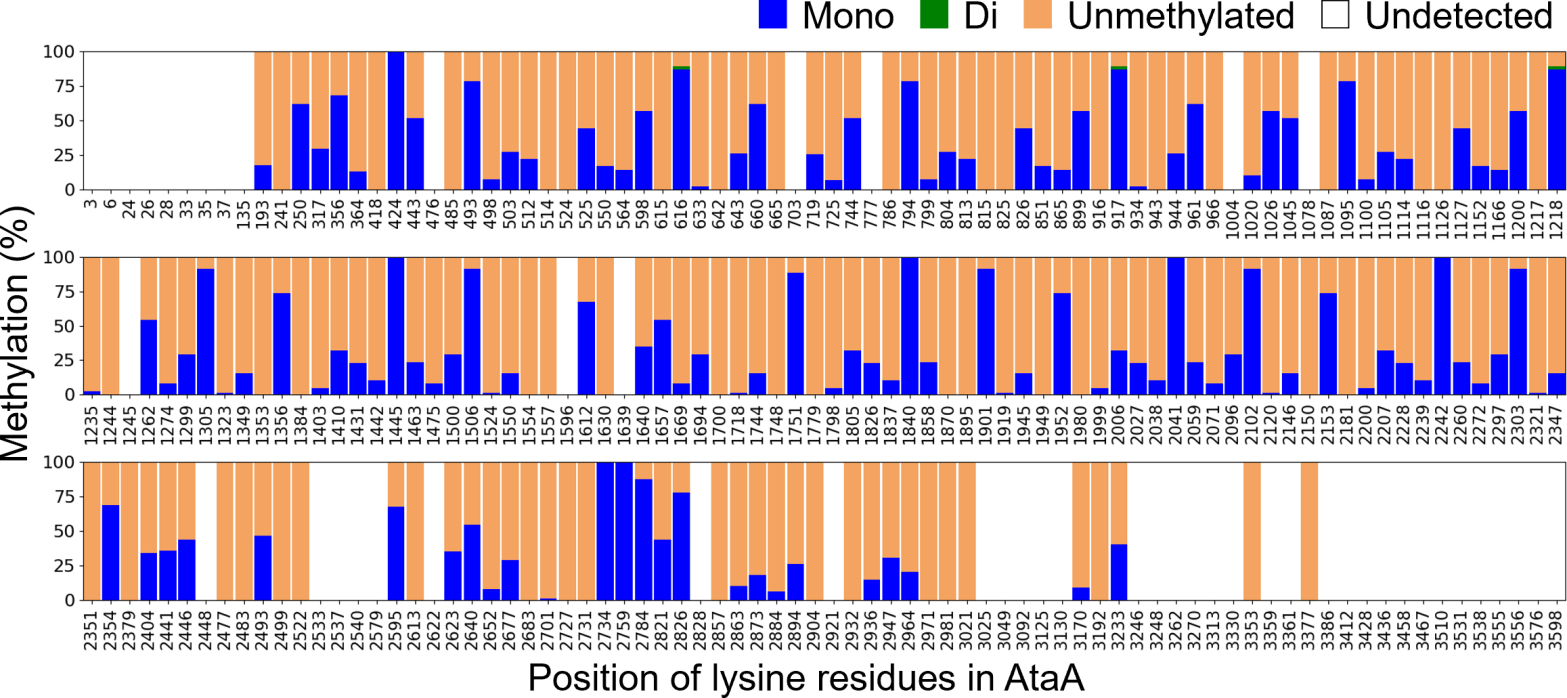
Lysine methylation of AtaA in *Acinetobacter* sp. Tol 5. The percentage of lysine residues that were unmethylated, monomethylated, or dimethylated. No trimethylated lysine residues were detected. The data are expressed as the means (*n* = 2).

In addition to the identification of cell surface fiber proteins, LC‒MS analysis revealed that the lysine residues of AtaA, the most abundant fiber protein on the cell surface of Tol 5, were methylated (Table S2). Although a few methylated lysine residues were identified in other proteins (Table S2), AtaA was unique in having more than 100 monomethylated lysine residues. The abundances of individual lysine residues and their methylation ratios were determined from the detected peptide fragments (Fig. 2; Tables S3 and S4). In the analysis, 183 of the 234 lysine residues in AtaA were detected, and methylation was detected at 131 of these residues. The predominant modification was monomethylation, where a single methyl group is transferred to the ε-amino group of a lysine residue. The monomethylated lysine residues were distributed throughout the AtaA polypeptide. However, dimethylation, where two methyl groups are added, was detected at only three residues (Lys616, Lys917, and Lys1218) and at low abundances. Trimethylated lysine residues were not detected.

### Identification of the enzyme responsible for the methylation of AtaA

**Fig 3:**
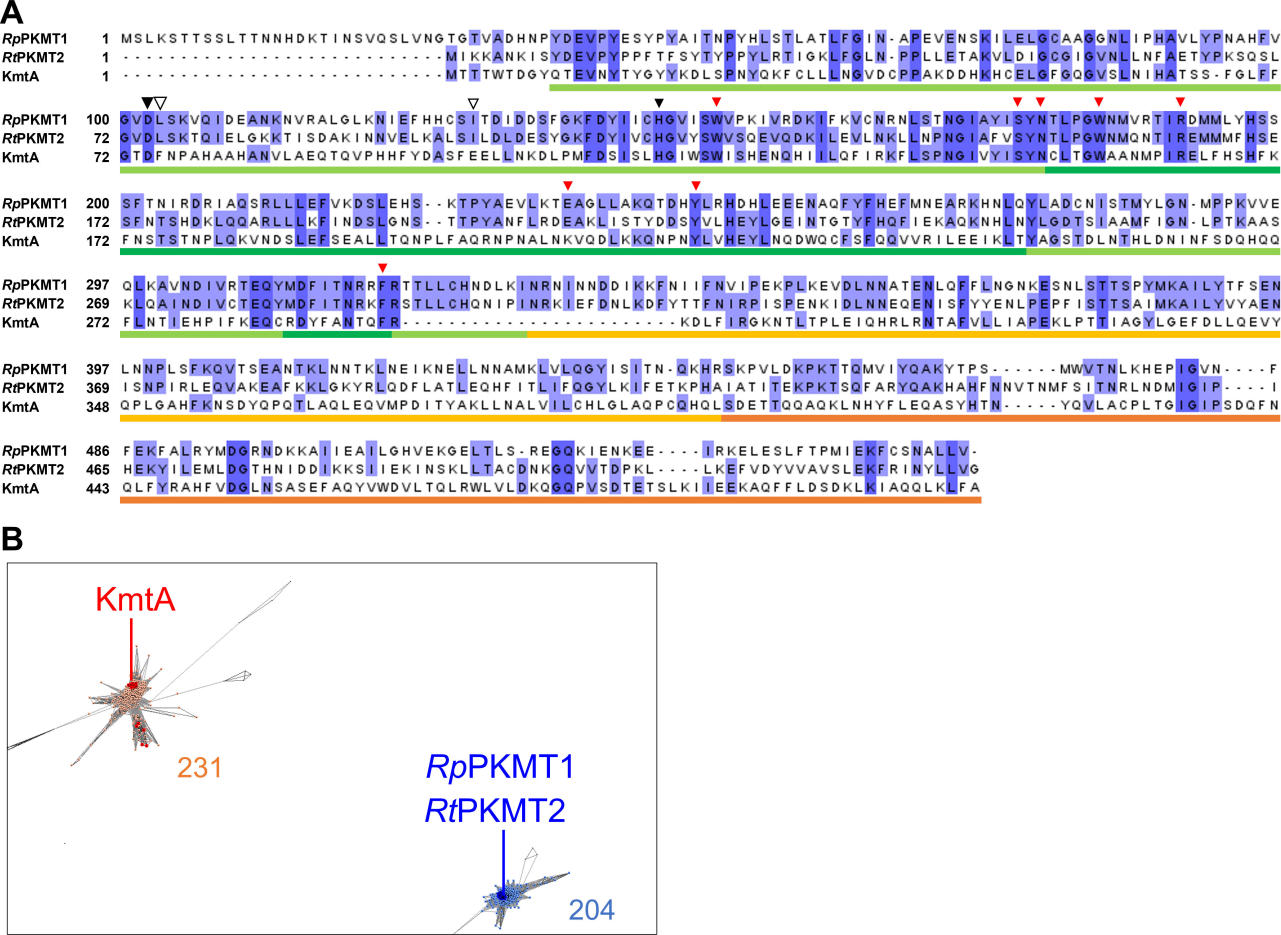
Amino acid sequence alignment and CLANS clustering analysis of *Rp*PKMT1, *Rt*PKMT2, and KmtA. (**A**) Multiple sequence alignment was obtained using ClustalW (28) and Jalview (29). Identical residues are highlighted in blue. The bars on the alignments show the structural domains of *Rp*PKMT1 and *Rt*PKMT2 (light green, AdoMet binding domain; green, dimerization domain; yellow, middle domain; and orange, C-terminal domain). The black triangles show the residues that form critical hydrogen bonds with AdoMet in *Rp*PKMT1. The white triangles show the residues at which the mutation to Ala affects the *K*_*m*_ value of *Rp*PKMT1 for binding to OmpB. Red triangles show the residues corresponding to the hydrophobic pocket of *Rp*PKMT1 and *Rt*PKMT2 that bind to OmpB. (**B**) Each dot represents amino acid sequences similar to that of KmtA, that is, OM PKMT. The cluster including KmtA (Cluster A) is indicated by the orange dots. The cluster including *Rp*PKMT1 and *Rt*PKMT2 (Cluster R) is indicated by light blue dots. Sequences from the reference genomes of *Acinetobacter* and *Rickettsia* are indicated by red and blue dots, respectively. Lines connecting the dots indicate the sequence similarity relationship at the BLAST *P* value cutoff of 10^−60^.

To identify the genes involved in the methylation of AtaA, whole-genome databases of Tol 5 (AP024708 and AP024709) were analyzed using BLAST. We found a gene encoding an amino acid sequence partially similar to that of OM PKMTs that were reported to methylate the OM protein OmpB in *Rickettsia* (26, 27). The amino acid sequence of the protein encoded by the identified gene (BCX72587.1) was aligned with those of *Rp*PKMT1 and *Rt*PKMT2 from *Rickettsia* (27) (Fig. 3A). The N-terminal region (Met1-Ile295) of the identified protein, including the methyl group-donor *S*-adenosylmethionine (AdoMet) binding domain (Tyr43-Asn179, Tyr261-Tyr286, and Arg318-Ile331) and the dimerization domain (Thr180-Phe260 and Leu287-Arg317) of *Rp*PKMT1, showed relatively high similarity (34% identity with conserved residues in *Rp*PKMT1 and *Rt*PKMT2), and the residues Asp102 and His146 in *Rp*PKMT1 (corresponding to Asp74 and His118 in *Rt*PKMT2), which form critical hydrogen bonds with AdoMet, were conserved in this new protein, strongly suggesting that it is a PKMT. This new PKMT was named KmtA. On the other hand, the C-terminal region (Asn296–Ala516) of KmtA, including the middle domain (Asn332–Arg447) and the C-terminal domain (Ser448–Val553) of *Rp*PKMT1, showed low similarity (16% identity with conserved residues in *Rp*PKMT1 and *Rt*PKMT2), and Leu103 and Ile130 in the AdoMet binding site of *Rp*PKMT1 (corresponding to Leu75 and Ile102 in *Rt*PKMT2), mutation of which to Ala affects the protein’s *K*_*m*_ value for binding to OmpB, were not conserved in KmtA.

The amino acid sequences with high similarity to that of KmtA were collected using PSI-BLAST and clustered based on sequence similarity using CLANS (30). As shown in Fig. 3B, two distinct clusters were observed: Cluster A contained KmtA, and Cluster R contained *Rp*PKMT1 and *Rt*PKMT2. While both clusters contained proteins from a wide range of species across phyla, some phyla were unique to each cluster (Table S5). For example, many species of Cyanobacteriota appeared only in Cluster A, while many species of PVC (Planctomycetes, Verrucomicrobia, and Chlamydiae) group bacteria appeared only in Cluster R. For Pseudomonadota (Proteobacteria), while sequences from the same order appeared in both clusters, certain sequences were unique to one cluster, such as those of Rhodobacterales and Cardiobacteriales in Cluster A. Note that the trends observed were based on the limited sequence information currently available. For the genera *Acinetobacter* and *Rickettsia*, homologous sequences were exclusively present in only the respective clusters (Fig. 3B). These results suggest that OM PKMTs with KmtA-like sequences are distributed in a wide range of bacterial species and are distinct from previously reported OM PKMTs, such as *Rp*PKMT1 and *Rt*PKMT2.

The type strains of *Acinetobacter baumannii*, ATCC 19606 and CCUG 19096, did not possess OM PKMTs, whereas other pathogenic *Acinetobacter* strains, such as *Acinetobacter haemolyticus* and *Acinetobacter junii,* had *kmtA*-like sequences in their genomes. Furthermore, complete genome sequences with genes encoding OM PKMT were collected and searched for TAA genes, revealing that 27 of 142 such genomic sequences also encode TAA genes (Table S6). Notably, bacteria such as *Burkholderia mallei*, *Burkholderia pseudomallei*, *Haemophilus influenzae*, and *Neisseria meningitidis*, known for their TAAs that mediate bacterial infection, also possess OM PKMT genes (31).

**Fig 4:**
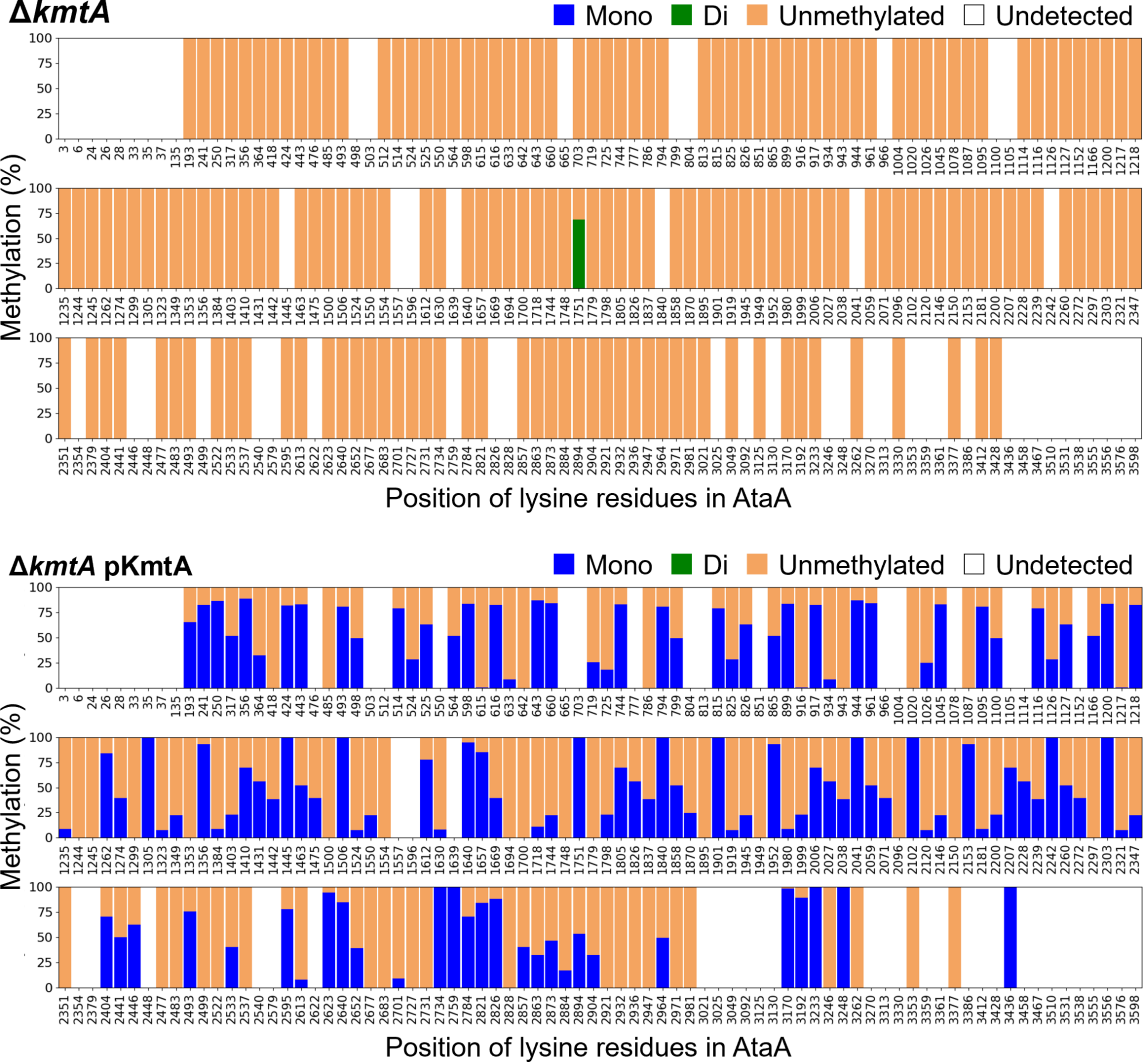
Lysine methylation of AtaA in the mutants of Tol 5. The percentage of lysine residues that were unmethylated, monomethylated, or dimethylated. No trimethylated lysine residues were detected. The data are expressed as the means (*n* = 2).

Lysine methylation of AtaA was examined in a *kmtA* gene knockout mutant strain of Tol 5 (Δ*kmtA*), which does not express KmtA due to point mutations introducing stop codons in the *kmtA* gene. In the LC‒MS analysis, only a single methylated lysine residue was detected in AtaA from Δ*kmtA* (Fig. 4; Tables S3 and S4). Furthermore, complementation of the *kmtA* gene (in the strain Δ*kmtA* pKmtA) restored the multiple lysine methylation of AtaA (Fig. 4; Tables S3 and S4). The average percentage of lysine methylation in AtaA from Δ*kmtA* pKmtA was greater (40%) than that in AtaA from the wild-type strain of Tol 5 (Tol 5 WT) (26%). These results indicate that KmtA is responsible for the multiple lysine methylation of AtaA.

In Tol 5 WT and Δ*kmtA*, a small number of proteins other than AtaA with mono-, di-, and trimethylated lysine residues were detected, including the ribosomal protein L11, which is known for multiple lysine methylation (32). In Δ*kmtA*, the total number of methylated lysine residues in proteins other than AtaA was similar to that in WT (Fig. S1; Table S2), suggesting that the methylation of these proteins is catalyzed by enzymes other than KmtA. In Δ*kmtA* pKmtA, an increase in the number of monomethylated proteins other than AtaA was observed. In this plasmid-complemented strain, the expression level of *kmtA* may have increased due to its increased copy number and enhanced methylation of lysine residues in both AtaA, the original substrate, and other proteins. In *Rickettsia*, multiple outer membrane proteins are methylated, and PKMT1 and PKMT2 are involved in the methylation of not only their primary substrate, OmpB, but also OmpA, ScaI, and OMP-porin (22). In contrast, Tol 5 possesses Omp38, which is homologous to OmpA, but no methylation of Omp38 was detected (Table S2). We aligned the amino acid sequences of AtaA up- and downstream of the detected methylated lysine residues to identify a potential consensus motif (±10 residues, Fig. S2). However, no clear consensus was found. Similarly, no consensus sequence has been reported for PKMTs in *Rickettsia*, or FilB, which methylates flagellin in *Salmonella* (23, 26).

### Effects of KmtA on the biogenesis of AtaA and cell physiology

**Fig 5:**
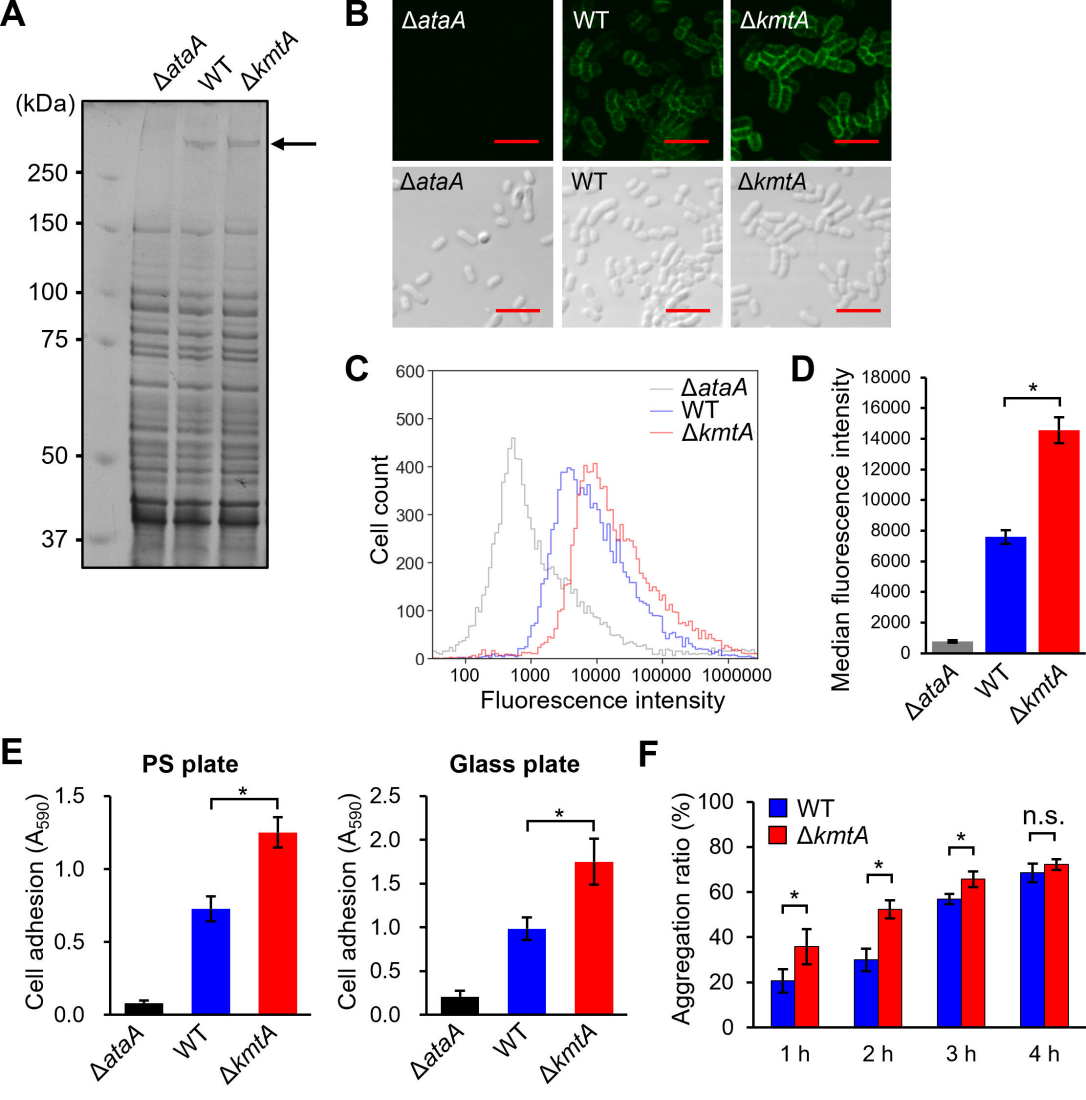
Effects of KmtA on the biogenesis of AtaA and cell physiology. (**A**) CBB staining of cell lysates of Tol 5 WT, Δ*ataA*, and Δ*kmtA* fractionated according to their molecular weight by SDS‒PAGE. The arrowhead shows the position of the AtaA bands. (**B**) Immunofluorescence microscopy of Tol 5 and its mutants using an anti-AtaA antiserum and an Alexa-Fluor 488-conjugated anti-rabbit antibody. Fluorescence and bright fields are shown. Scale bar: 5 µm. (**C**) Fluorescence flow cytometry analysis of Tol 5 and its mutants using an anti-AtaA antiserum and an Alexa-Fluor 488-conjugated anti-rabbit antibody. (**D**) The mean fluorescent intensity values, based on the medians from three independent flow cytometry experiments, are presented as mean ± SD (*n* = 3). Statistical significance, *P < 0.01. (**E**) Adherence assay of Tol 5 WT, Δ*ataA*, and Δ*kmtA* using 96-well plates made of polystyrene (PS) and glass. The data are expressed as the mean ± SD (*n* = 5). Statistical significance, *P < 0.01. (**F**) Autoagglutination assay of Tol 5 WT and Δ*kmtA*. The graph bars show the autoagglutination ratio (%) at each time point. The data are expressed as the mean ± SD (*n* = 3). Statistical significance, *P < 0.05; n.s., not significant.

We next investigated whether lysine methylation affects the biogenesis of AtaA fibers in Tol 5. To examine the production of the AtaA protein, whole-cell lysates of WT, Δ*ataA*, and Δ*kmtA* were separated by sodium dodecyl sulfate‒polyacrylamide gel electrophoresis (SDS‒PAGE). On the gel stained with Coomassie brilliant blue (CBB), AtaA polypeptides from WT and Δ*kmtA* exhibited almost the same signal intensity and electrophoretic mobility (Fig. 5A). Considering that the addition of a single methyl group to all 234 lysine residues of AtaA increases its molecular weight by only 3.1 kDa, it is reasonable that methylation of AtaA, with a molecular weight greater than 300 kDa, would hardly change its electrophoretic mobility. The similar band intensity between WT and Δ*kmtA* indicates that the disruption of *kmtA* did not affect the overall production of AtaA. To examine the cell surface display of AtaA, WT, Δ*ataA*, and Δ*kmtA* were stained with anti-AtaA antiserum and Alexa 488-conjugated anti-rabbit antibody and then subjected to confocal laser scanning microscopy and flow cytometry (Fig. 5B through D; Fig. S3). Both WT and Δ*kmtA* exhibited fluorescence, but the intensity of the fluorescence in Δ*kmtA* was greater than that in WT. As the methylation of lysine residues in AtaA by KmtA did not distinctly affect the recognition by anti-AtaA antiserum (Fig. S4), the increased fluorescence intensity in Δ*kmtA* indicates that the disruption of *kmtA* enhanced the cell surface display of AtaA.

Subsequently, we examined the adhesiveness and autoagglutination of Δ*kmtA*. This mutant strain showed higher adhesiveness to both hydrophobic polystyrene (PS) and hydrophilic glass plates than did WT (Fig. 5E). While both WT and Δ*kmtA* exhibited autoagglutination, the autoagglutination process of Δ*kmtA* was faster than that of WT (Fig. 5F). These results suggest that the KmtA-mediated methylation of AtaA reduces the amount of AtaA on the Tol 5 cell surface, resulting in a decrease in the adhesiveness and autoagglutination of Tol 5 cells.

**Fig 6:**
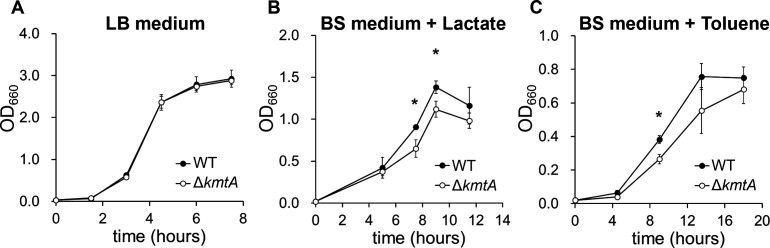
Growth of *Acinetobacter* sp. Tol 5 WT and Δ*kmtA*. (**A**) Tol 5 WT and Δ*kmtA* were grown in LB medium. The optical density at 660 nm (OD_660_) of the cell culture was measured. The data are expressed as the mean ± SD (biological replicate *n* = 3). (**B and C**) Tol 5 WT and Δ*kmtA* were grown in BS medium supplemented with lactate or toluene as a carbon source. Polyurethane foam was used as a carrier for cell adhesion. Cells adhering to the polyurethane foam were detached using casamino acids followed by mixing prior to each OD_660_ measurement. The data are expressed as the mean ± SD (biological replicate *n* = 3). Statistical significance, *P < 0.05.

We further examined the effect of *kmtA* gene disruption on the growth of Tol 5 cells under several conditions. In Luria–Bertani (LB) medium, which inhibits the adhesion and autoagglutination of Tol 5 cells mediated by AtaA (33), WT and Δ*kmtA* exhibited similar growth curves (Fig. 6A). In basal salt (BS) medium supplemented with lactate or toluene as the sole carbon source, where Tol 5 cells exhibit high adhesiveness and autoagglutination mediated by AtaA, the growth rate of Δ*kmtA* was lower than that of WT (Fig. 6B and C). In addition, the double-knockout mutant of *ataA* and *kmtA* of Tol 5 (Δ*ataA*Δ*kmtA*) showed no significant change in growth rate compared with Δ*ataA* in either LB or BS medium (Fig. S5). These results indicate that KmtA promotes the growth of Tol 5 through the methylation of AtaA in environments where cells exhibit adhesiveness and autoagglutination by AtaA.

## DISCUSSION

Protein lysine methylation is a common posttranslational modification that occurs in histones and various nonhistone proteins. Lysine methylation plays a crucial role in diverse biological processes, such as the regulation of chromatin conformation, signal transduction, the DNA damage response, protein folding, metabolism, and cell growth in eukaryotes (19–21). Recent studies have revealed that methylation of cell surface proteins or secreted proteins in pathogenic bacteria contributes to their infectivity through various mechanisms (22, 23, 34). For example, multiple lysine methylation of OmpB prevents ubiquitination and subsequent autophagy of *Rickettsia*, while multiple lysine methylation of *Salmonella* flagellin enhances flagellar hydrophobicity, promoting host cell invasion. However, most studies have focused on histones in eukaryotes, and the biological functions of lysine methylation in bacteria remain largely unknown, even though lysine methylation was first discovered in bacteria (21, 35). In this study, we conducted proteome analysis of Tol 5 and coincidentally found the multiple lysine methylation of AtaA (Tables S2 and S3). This is the first report demonstrating the posttranslational multiple lysine methylation of TAAs, describing the most extensively methylated protein among reported methylated bacterial OM proteins. Further experiments with Tol 5 Δ*kmtA* revealed that KmtA is responsible for the methylation of AtaA and has a role in controlling the amount of AtaA displayed on the cell surface and the adhesion and agglutination nature of Tol 5 cells, contributing to enhanced growth.

In *Acinetobacter baumannii*, a comprehensive analysis of lysine trimethylation was conducted using strong cation exchange chromatography (SCX) fractionation and multiple software platforms for MS/MS spectra analysis (36). This revealed that various proteins, including membrane proteins such as ABC transporters and multi-drug efflux pumps, undergo trimethylation at one to three lysine residues. Despite the lack of the *kmtA* gene in *A. baumannii*, trimethylation has been observed at one site in its TAA family protein Ata. In contrast, our analysis of Tol 5 revealed extensive monomethylation at over 130 lysine residues in AtaA. In the Δ*kmtA* mutant of Tol 5, while monomethylation was entirely absent, one dimethylated lysine residue was still detected in AtaA (Fig. 4), suggesting the presence of other methyltransferases that may methylate TAAs in *Acinetobacter* species. However, since no enrichment steps specifically targeting modified peptides were employed in our analysis, it is possible that additional methylated lysine residues were not detected. The use of enrichment techniques, such as SCX fractionation and affinity-based methods utilizing pan-methyllysine antibodies, could lead to a more comprehensive identification of methylated lysine residues (36, 37).

The suppression of the growth of Δ*kmtA* was observed only when BS medium was used for cultivation, while no difference was observed in LB medium (Fig. 6A through C). In a previous study, we reported that the adhesion and autoagglutination of Tol 5 through AtaA were inhibited in LB medium (33). Given that adherent or agglutinating cells often exhibit slower growth than planktonic cells due to the restricted transport and diffusion of cells and substrates (38), the high adhesiveness and autoagglutination of Δ*kmtA* may have resulted in slower growth in BS medium, where Tol 5 exhibits high adhesiveness and autoagglutination; this suggests that methylation of AtaA indirectly promotes Tol 5 cell growth by decreasing the amount of AtaA on the cell surface. In fact, we previously reported that Tol 5 displays AtaA fibers poorly in the early growth phase for efficient and fast cell growth (39).

OM proteins other than AtaA in Tol 5 were barely methylated (Table S2), indicating that methylation by KmtA is more specific than that by previously reported rickettsial PKMTs, which methylate not only the main target OmpB but also other OM proteins, such as OmpA and Sca1 (22). Seven of the eight amino acid residues corresponding to the hydrophobic pocket of *Rp*PKMT1 and *Rt*PKMT2 that bind to OmpB were conserved in KmtA (27). On the other hand, L103 and I130 near the AdoMet binding site, which are important for OmpB recognition by *Rp*PKMT1 and *Rt*PKMT2, were not conserved in KmtA. These findings suggest that the substrate recognition mechanism of KmtA is somewhat different from that of rickettsial PKMTs. Clustering analysis further revealed that OM PKMTs with KmtA-like sequences form a distinct cluster separate from rickettsial PKMTs. Comparison of multiple sequence alignments of amino acid sequences derived from each cluster revealed distinct differences between sequences in Clusters A and R (Fig. S6). Notably, the conserved motif of charged amino acids in the N-terminal region of Cluster A proteins (Lys282–Arg295 in KmtA) markedly differs from that in Cluster R proteins. Because this region is located within the cleft predicted to bind substrate peptides, according to the working model based on crystal structure analyses of rickettsial PKMTs, it may play a significant role in the interaction with the substrate. The C-terminal region of OM PKMTs contains several subregions that share high sequence similarity within each cluster (Fig. S6). These features suggest that there are two distinct subfamilies of OM PKMTs, for which we propose the names OM PKMT A, which includes *Acinetobacter* KmtA, and OM PKMT R, which includes *Rp*PKMT1 and *Rt*PKMT2.

TAAs from pathogenic bacteria have diverse functions, such as adhesion, autoagglutination, biofilm formation, serum resistance, and actin-based motility, and are crucial for infection (40–42). In this study, OM PKMTs were identified in a wide range of bacteria, and 19% of them also possessed TAAs (Table S6). In these bacteria, TAAs may be methylated, similar to AtaA, and this methylation may be involved in diverse functions related to their pathogenicity and biofilm formation. Especially, it is possible that KmtA-mediated lysine methylation of TAAs contributes to preventing ubiquitination and subsequent autophagy just as rickettsial PKMT-mediated lysine methylation does in *Rickettsia* (22). However, some species possess OM PKMTs but not TAAs; OM PKMTs in these species may methylate other OM proteins and contribute to their biological functions, such as growth or infectivity.

In conclusion, we discovered multiple lysine methylation on AtaA and identified the enzyme responsible for this methylation. This study constitutes the first report of TAA methylation. Our findings contribute to a better understanding of posttranslational modifications in bacteria, which is important for both medical and engineering applications.

## MATERIALS AND METHODS

### Sequence analysis

Amino acid sequence alignments were generated using ClustalW (28) and Jalview version 2.11.3.2 (29). To collect sequences with high similarity to KmtA, five iterative PSI-BLAST searches (43) were performed against the uniprot_trembl database (accessed on 20 January 2024) at an *E*-value cutoff of 10^−60^. After the highly redundant sequences were removed using HHfilter (43) at a 50% identity cutoff, the sequences with genus-level annotations were extracted. Sequences with high similarity to KmtA were also collected from NCBI reference genomes (Assembly level: complete) of *Acinetobacter* species and *Rickettsia* species using BLASTP at an *E*-value cutoff of 10^−10^. These sequences were also analyzed using CLANS (30) at an *E*-value cutoff of 10^−60^. The sequences used in the CLANS analysis were reduced using HHfilter at a 25% identity cutoff and searched against the RefSeq representative genome database of bacteria using tBLASTn (https://blast.ncbi.nlm.nih.gov/Blast.cgi) to collect complete genome sequences containing sequences encoding PKMT. Then, the amino acid sequences of the transmembrane domain of TAAs reported by Thibau et al. (31) were searched against the collected complete genome sequences.

### Bacterial strains and growth conditions

The primers, bacterial strains, and plasmids used in this study are shown in Tables S7 and S8. *Acinetobacter* sp. Tol 5 and its derivative mutants were grown in BS medium supplemented with toluene or LB medium at 28°C as described previously (44). *Escherichia coli* was grown in LB medium at 37°C. Apramycin (100 µg/mL) and gentamicin (10 µg/mL) were used as needed. Arabinose was added at a final concentration of 0.5% (wt/vol) for the induction of *kmtA* expression under the control of the P_BAD_ promoter.

To obtain growth curves, 2 mL of the overnight culture of Tol 5 and its mutants in LB medium was centrifuged (5,000 × *g*, 25°C, 5 min), washed twice with BS medium, and resuspended in BS medium. Twenty microliters of the suspensions was inoculated into 20-mL vials (Maruemu Corporation, Osaka, Japan) containing 2 mL of BS medium or 2 mL of LB medium with four pieces of polyurethane foam support with a specific surface area of 37.5 cm^2^/cm^3^ (CFH-30; Inoac Corporation, Nagoya, Japan) in the shape of a rectangle (10 × 5 × 5 mm). As a carbon source, 8 µL of 50% sodium DL-lactate or 2.5 µL of toluene was added to the medium, and the vials were incubated at 28°C with shaking at 115 rpm. At each time point during cultivation, three vials were collected, and the cultures were mixed vigorously with 100 µL of 10% Casamino Acids technical grade (CA-T; Becton, Dickinson and Company, Franklin Lakes, NJ, USA) to detach the bacterial cells from the support (33). The OD_660_ of the cell suspension was measured by a UV‒Vis spectrophotometer (UV-1850; Shimadzu Corporation, Kyoto, Japan).

### Plasmid construction and gene knockout

The *kmtA* gene knockout mutant (Δ*kmtA*) was generated using the cytidine base editing system for *Acinetobacter* (45). The inserted DNA dimer, encoding the sequence of the single guide RNA, was prepared by mixing oligo DNAs (kmtA-spacer-BsaI-F and kmtA-spacer-BsaI-R) in 50 mM NaCl solution and gradually cooling from 95°C to 18°C at 0.1°C/s. This dimer was introduced into pBECAb-apr using the NEBridge Golden Gate Assembly Kit (New England Biolabs, Ipswich, MA, USA) to generate pBECAb-apr-kmtA. Transformation of Tol 5 and its mutant was performed through REK123Δ*ataA*, following the method previously reported (46). After overnight culture of Tol 5 and its mutant harboring pBECAb-apr-kmtA in BS medium to promote mutation, the cells were spread on a BS agar plate containing 5% (wt/vol) sucrose. Mutation of the *kmtA* gene in the resultant colonies was confirmed by sequencing the DNA from the colonies via PCR using KOD FX Neo (TOYOBO, Osaka, Japan).

To construct pKmtA, a fragment containing the deduced Shine‒Dalgarno sequence and the entire *kmtA* gene was amplified by colony PCR from Tol 5 using the primers ARP3kmtAF and ARP3kmtAR. The PCR product was inserted into the EcoRI-XbaI site of pARP3, generating pKmtA. Transformation of Δ*kmtA* was carried out by bacterial conjugation with the donor strain *E. coli* S17-1 harboring pKmtA as previously described (14).

### Sample preparation for mass spectrometry

Sample preparation for LC‒MS analysis was performed as described previously (22). One milliliter of cell suspension of Tol 5 and its mutants grown in LB medium was transferred to a 1.5-mL microtube and centrifuged (5,000 × *g*, 4°C, 5 min) to collect the precipitated bacterial cells. The cells were resuspended in 333 µL of Tris-EDTA solution (10 mM Tris-HCl and 10 mM EDTA, pH 7.6), incubated at 45°C for 90 min, and then boiled at 95°C for 10 min. After cooling to room temperature, 20 µL of 50 mM NH_4_HCO_3_ (pH 7.5) and 50 µL of 0.2% (wt/vol) RapiGest (Waters Corporation, Milford, MA, USA) diluted in 50 mM NH_4_HCO_3_ were added to 50 µL of the samples and heated at 80°C for 15 min. After cooling to room temperature, the samples were digested with 1 µg of Pierce Trypsin Protease (Thermo Fisher Scientific, Waltham, MA, USA) at 37°C overnight. To hydrolyze the RapiGest, 20 µL of 5% (vol/vol) trifluoroacetic acid was added to the samples, which were then incubated at 37°C for 90 min. After centrifugation (15,000 × *g*, 4°C, 25 min), the samples were desalted using ZipTip (ZTC18M096; Merck Millipore, Burlington, MA, USA) and dried using a CVE-3100 (Tokyo Rikakikai Co., Ltd., Tokyo, Japan).

### Liquid chromatography–mass spectrometry

The digested peptides were analyzed by nanoflow reverse-phase LC followed by tandem MS using a Q Exactive hybrid mass spectrometer (Thermo Fisher Scientific). The capillary reverse-phase HPLC-MS/MS system was composed of a Dionex U3000 gradient pump equipped with a VICI CHEMINERT valve. The Q Exactive instrument was equipped with a nanoelectrospray ionization source (AMR). The desalted peptides were loaded into a separation capillary C18 reverse-phase column (NTCC-360/100-3-125, 125 × 0.1 mm; Nikkyo Technos Co., Ltd., Tokyo, Japan). The peptide spectra were recorded over the mass range of *m*/*z* 350–1,800 using the Xcalibur 4.1.50 system (Thermo Fisher Scientific). MS spectra were recorded repeatedly, followed by 20 data-dependent high-energy collisional dissociation MS/MS spectra generated from the 10 highest-intensity precursor ions. MS/MS spectra were interpreted, and peak lists were generated using Proteome Discoverer 2.4.1.15 (Thermo Fisher Scientific). Searches against the *Acinetobacter* sp. Tol 5 whole-genome database (AP024708 and AP024709; NCBI) were performed using SEQUEST (Thermo Fisher Scientific). The search parameters were set as follows: enzyme selected with two maximum missed cleavage sites, a mass tolerance of 10 ppm for peptide tolerance and 0.02 Da for MS/MS tolerance, and oxidation (M), monomethylation (R, K), dimethylation (R, K), trimethylation (R, K), and N-terminal acetylation as variable modifications. Peptide identification was based on significant Xcorr values (high confidence filter). Peptide abundance was calculated on the basis of the sum of the peptide peak areas derived from label-free quantification using Proteome Discoverer software. The abundance of individual lysine residues in AtaA was calculated as the sum of the abundance of the peptides containing these residues, excluding peptides with detected modifications of ambiguous localization. For the peptides whose amino acid sequences appeared repeatedly at multiple positions in AtaA, the abundance value was divided by the number of repeats and assigned equally to all of those positions. The percentage of lysine methylation (mono-, di-, tri-, or unmethylated) was calculated by dividing the abundance of each methylated residue by the total abundance of the residue and multiplying by 100.

### Polyacrylamide gel electrophoresis

One milliliter of cell suspension of Tol 5 or its mutants was transferred to a 1.5-mL microtube and centrifuged (5,000 × *g*, 4°C, 5 min). The precipitated cells were resuspended in SDS‒PAGE sample buffer (5% [vol/vol] 2-mercaptoethanol, 2% [wt/vol] SDS, 0.02% [wt/vol] bromophenol blue, and 62.5 mM Tris-HCl, pH 6.8) to adjust the OD_660_ to 10, and boiled at 100°C for 10 min. After diluting the samples 10-fold for western blotting, they were analyzed by SDS‒PAGE using a 7.5% polyacrylamide gel, followed by staining with CBB or western blotting using anti-AtaA_699-1014_ antiserum (44).

### Immunofluorescence microscopy

Immunofluorescence microscopy was performed as described previously (44) with slight modifications. Twenty microliters of bacterial cell culture was placed on a glass slide (TF1205M; Matsunami Glass Ind., Ltd., Osaka, Japan) and incubated at room temperature for 15 min. The cells were fixed with an equal volume of 4% paraformaldehyde, incubated at room temperature for 15 min, and washed twice with phosphate-buffered saline containing Tween 20 (PBS-T; 1.47 mM KH_2_PO_4_, 8.1 mM Na_2_HPO_4_, 137 mM NaCl, 2.68 mM KCl, and 0.05% [wt/vol] Tween 20, pH 7.4). The samples were incubated with anti-AtaA_699-1014_ antiserum (44) at a 1:10,000 dilution in PBS-T at room temperature for 30 min. The samples were washed twice with PBS-T and incubated with an AF488-conjugated anti-rabbit antibody (Cell Signaling Technology, Inc., Danvers, MA, USA) at a 1:1,000 dilution in PBS-T at room temperature for 30 min. The samples were washed three times with PBS-T and once with pure water and observed under a confocal laser scanning fluorescence microscope (FV1000; Olympus, Tokyo, Japan). Images were obtained with UPLSAPO100XO objective lens (Olympus, Japan). The excitation/emission wavelength of 473 and 485–530 nm was used for Alexa Fluor 488. The images were visualized using image analysis software (FV10-ASW [version 1.7]). The same exposure settings and brightness parameters were applied across all samples.

### Flow cytometry

Flow cytometry was performed as described previously (14) with slight modifications. One milliliter of bacterial culture was transferred to a 1.5-mL microtube and centrifuged (5,000 × *g*, 4°C, 5 min). The precipitated cells were then fixed with 4% paraformaldehyde at room temperature for 15 min. After washing twice with PBS-T containing 1% CA-T, the samples were incubated with anti-AtaA_699-1014_ antiserum at a 1:10,000 dilution in PBS-T containing 1% CA-T at 4°C for 1 h with inversion mixing. The samples were washed twice with PBS-T containing 1% CA-T and incubated with an AF488-conjugated anti-rabbit antibody at a 1:1,000 dilution in PBS-T containing 1% CA-T at room temperature for 1 h with inversion mixing. The immunostained samples were washed in PBS-T containing 1% CA-T and resuspended in 500 µL of PBS-T, and fluorescence was measured using a flow cytometer (CytoFLEX S; Beckman Coulter, Brea, CA, USA).

### Bacterial adhesion and autoagglutination assay

Adherence assays and autoagglutination assays were performed as previously described (39) with slight modifications. For the adherence assay, cell suspensions of Tol 5 and its mutants were placed into a 96-well PS microplate (353072; Becton, Dickinson and Company) and a 96-well glass plate (FB-96; Nippon Sheet Glass Co., Ltd., Tokyo, Japan), followed by incubation at 28°C for 2 h. The adhered cells were stained with 0.1% crystal violet, and the stain was eluted from the cells with 200 µL of 70% ethanol. The absorbance at 590 nm (*A*_590_) of the eluted solution was measured by a microplate reader (ARVO V3; PerkinElmer, Waltham, MA, USA). In the autoagglutination assay, test tubes (TEST13-100NP, Iwaki, Tokyo, Japan) containing 8 mL of cell suspension were left at room temperature for sedimentation of the cell clumps generated by autoagglutination. The autoagglutination ratio was calculated based on the decrease in the OD_660_ value of the cell suspension due to cell sedimentation using the following equation: Autoagglutination ratio (%) = 100 × (initial OD_660_ − OD_660_ after standing)/initial OD_660_.

### Statistical analysis

Error bars denote the standard deviation of the mean derived from at least three independent biological replicates. Standard deviations were calculated and analyzed using a two-tailed Student’s *t* test with Excel. Data differences were considered significant if *P ≤ 0.05.

## Data Availability

Mass spectrometry proteomics data were deposited to jPOSTrepo (47) with the data set identifiers JPST003124 and PXD052460.

## SUPPLEMENTAL MATERIAL

Fig. S1 to S6, Tables S7 to S8 jb.00325-24-s0001.pdf Fig. S1 to S6, Tables S7 to S8.

Table S1 jb.00325-24-s0002.xlsx Label-free LC‒MS analysis of Tol 5.

Table S2 jb.00325-24-s0003.xlsx Peptides with methylation.

Table S3 jb.00325-24-s0004.xlsx Peptide fragments from AtaA.

Table S4 jb.00325-24-s0005.xlsx Lysine residues in AtaA.

Table S5 jb.00325-24-s0006.xlsx Genera found in the two clusters in Figure 3B.

Table S6 jb.00325-24-s0007.xlsx Bacterial strains.

ASM does not own the copyrights to Supplemental Material that may be linked to, or accessed through, an article. The authors have granted ASM a non-exclusive, world-wide license to publish the Supplemental Material files. Please contact the corresponding author directly for reuse.
