# Supplementary material for: A new target of multiple lysine methylation in bacteria
Source: J Bacteriol. 2024 Dec 11;207(1):e00325-24. doi: 10.1128/jb.00325-24 (PMC11784438; doi:10.1128/jb.00325-24)
Supplement: Fig. S1 to S6, Tables S7 to S8 [file jb.00325-24-s0001.pdf]

Supporting Information for

A new target of multiple lysine methylation in bacteria

Shori Inoue, Shogo Yoshimoto, and Katsutoshi Hori[#]

Department of Biomolecular Engineering, Graduate School of Engineering, Nagoya University, Furo-cho, Chikusa-ku, Nagoya, Aichi 464-8603, Japan

# Correspondence: Katsutoshi Hori
Department of Biomolecular Engineering, Graduate School of Engineering, Nagoya University, Furo-cho, Chikusa-ku, Nagoya, Aichi 464-8603, Japan.
Tel: +81-52-789-3339
Email: khori@chembio.nagoya-u.ac.jp

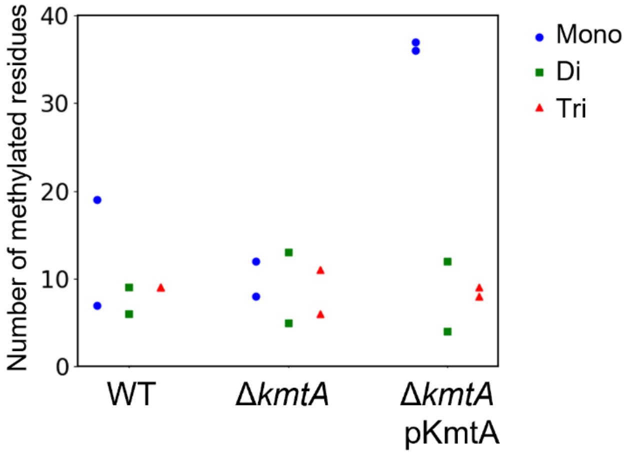

Figure S1. The number of methylated lysine residues, excluding AtaA residues. Count of methylated lysine residues (mono-, di-, and tri-) in peptides quantified by LC–MS, in which the peptides from AtaA were excluded (biological replicates, n = 2).

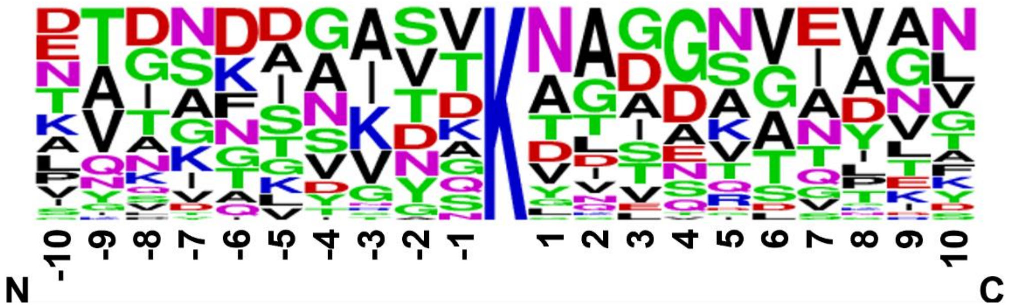

Figure S2. Amino acid frequencies of peptide sequences around lysine residues with over 50% methylation ratio in AtaA. WebLogo (https://weblogo.berkeley.edu/logo.cgi) was used to analyze the conservation of the peptide sequences (methylated lysine ± 10 residues).

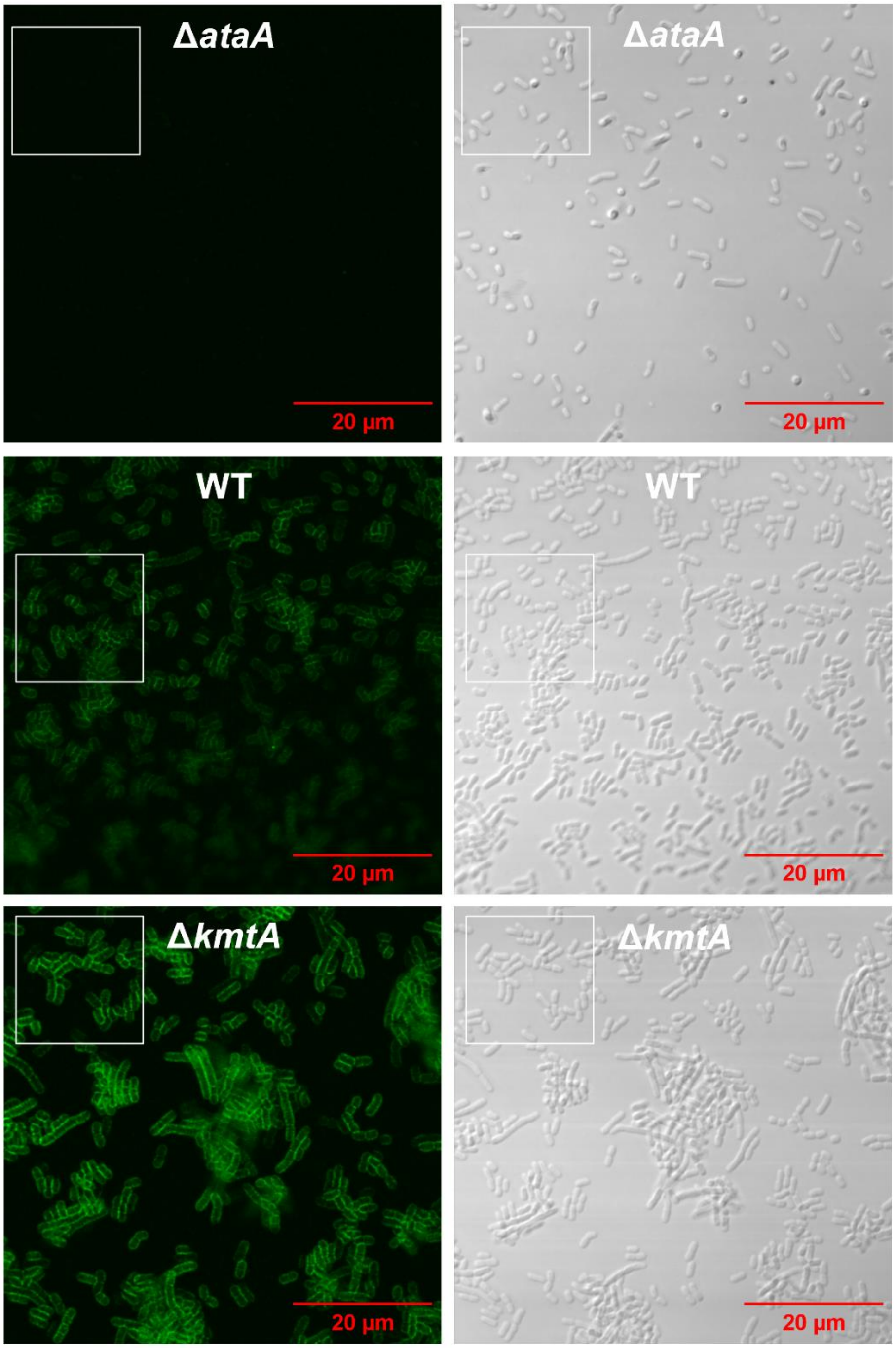

Figure S3. Confocal laser scanning microscopy (CLSM) images of Tol 5 and its mutants. Bacteria were stained using an anti-AtaA antiserum and an Alexa-Fluor 488-conjugated anti-rabbit antibody. Fluorescence and bright fields are shown. The regions outlined by white solid lines were shown in Figure 5B.

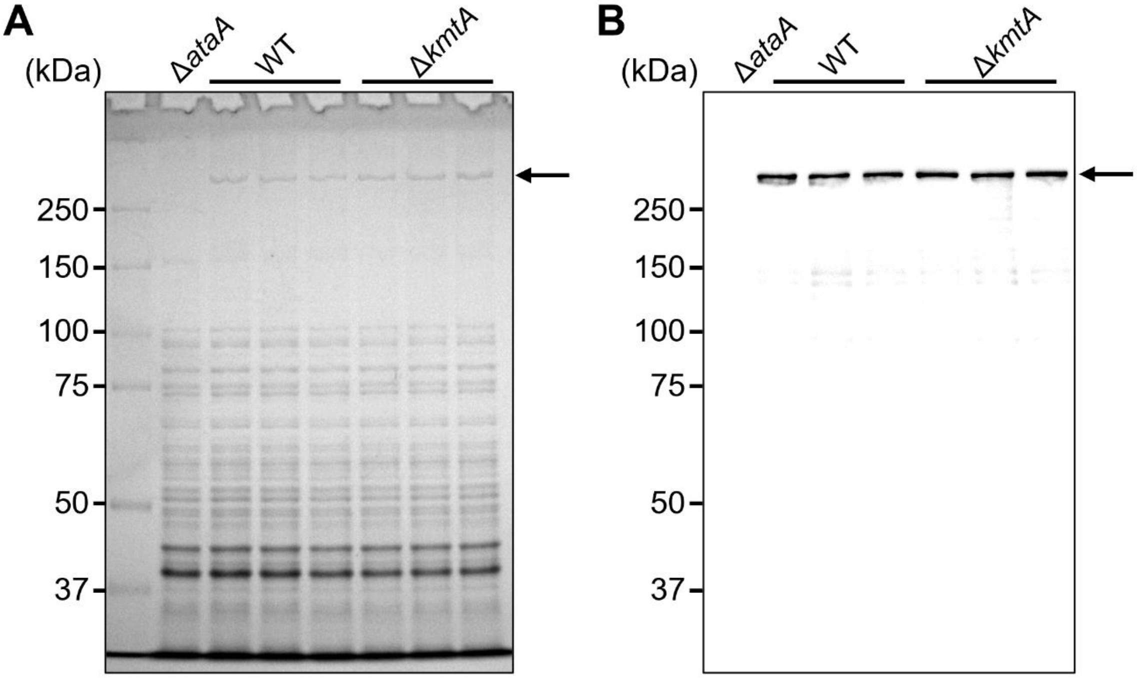

Figure S4. Expression of AtaA in Tol 5 WT, Δ*ataA*, and Δ*kmtA*. (A) CBB staining and (B) western blot analysis of proteins from cell lysates of Tol 5 WT, Δ*ataA*, and Δ*kmtA*, fractionated by SDS-PAGE based on molecular weight. For the western blotting, samples were diluted 10-fold and immunostained using anti-AtaA antiserum. The arrowhead indicates the position of the AtaA bands.

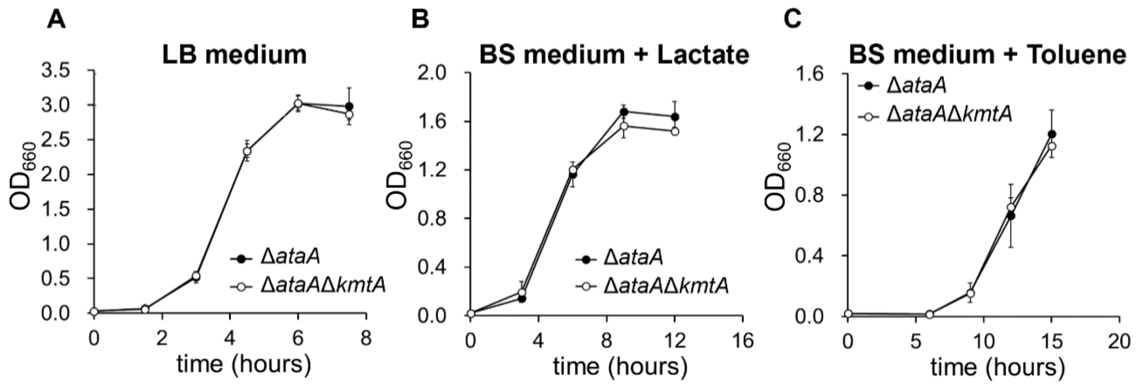

Figure S5. Growth of *Acinetobacter* sp. Tol 5 Δ*ataA* and Δ*ataA*Δ*kmtA*. (A) Δ*ataA* and Δ*ataA*Δ*kmtA* were grown in LB medium. The optical density at 660 nm (OD_{660}) of the cell culture was measured. The data are expressed as the mean ± SD (biological replicate n = 3). (B, C) Tol 5 Δ*ataA* and Δ*ataA*Δ*kmtA* were grown in BS medium supplemented with lactate or toluene. Polyurethane foam was used as a carrier for cell adhesion. Cells adhering to the polyurethane foam were detached using casamino acid followed by mixing prior to each OD_{660} measurement. The data are expressed as the mean ± SD (biological replicate n = 3).

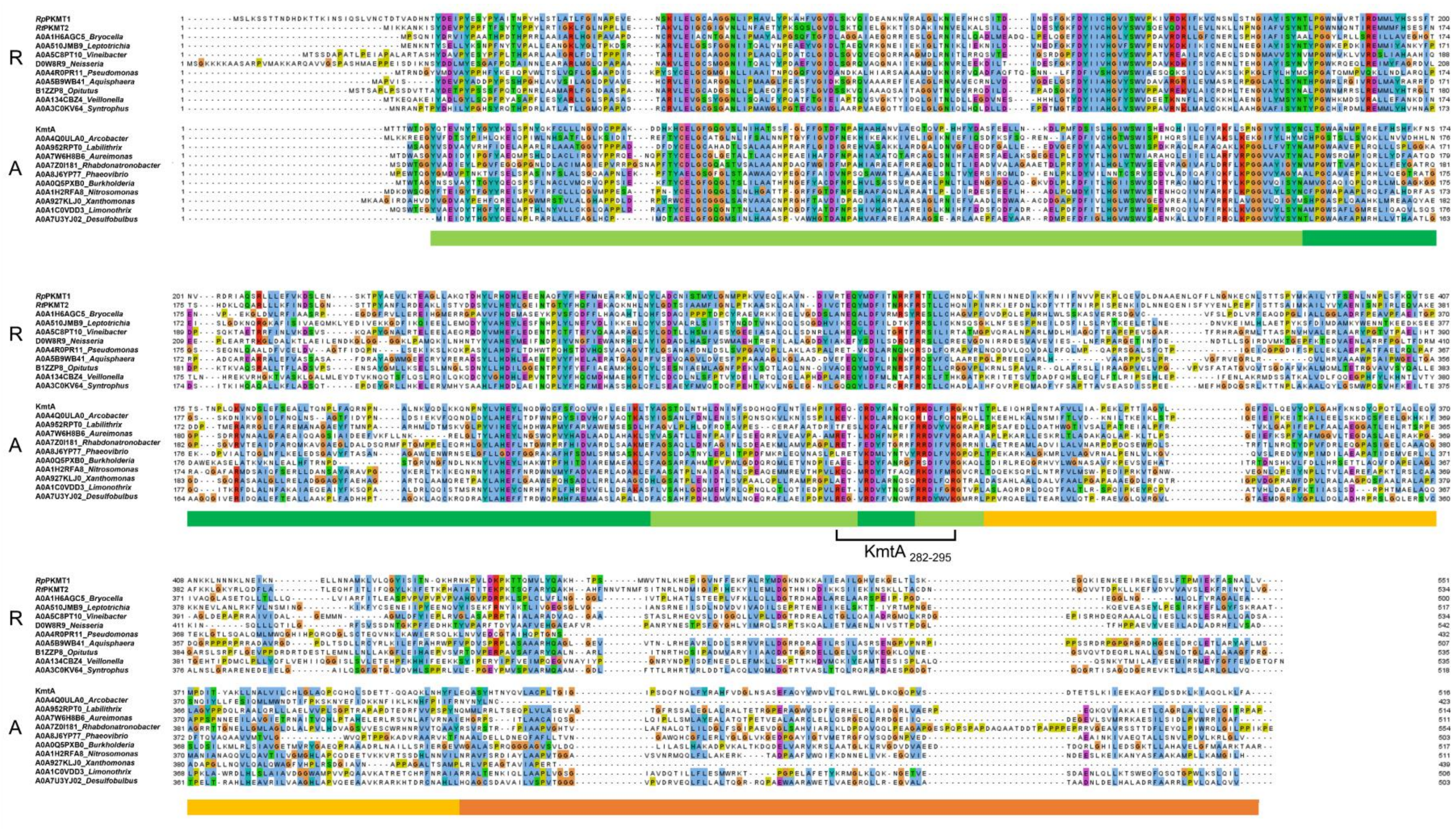

R
A
RpPKMT1
RfPKMT2
A0A1H6AGC5_Bryocella
A0A510JMB9_Leptotrichia
A0A5C8PT10_Vineibacter
D0W8R9_Neisseria
A0A4R0PR11_Pseudomonas
A0A5B9WB41_Aquisphaera
B1ZZP8_Opitutus
A0A134CBZ4_Veillonella
A0A3C0KV64_Syntrophus
KmtA
A0A4Q0ULA0_Arcobacter
A0A952RPT0_Labilithrix
A0A7W6H8B6_Aureimonas
A0A7Z0I181_Rhabdonatronobacter
A0A8J6YP77_Phaeovibrio
A0A0Q5PXB0_Burkholderia
A0A1H2RFA8_Nitrosomonas
A0A927KLJ0_Xanthomonas
A0A1C0VDD3_Limonothrix
A0A7U3YJ02_Desulfobulbus
KmtA 282-295

Figure S6. A comparison of multiple sequence alignments of amino acid sequences derived from Cluster A and Cluster R shown in Figure 3B. Multiple sequence alignments (MSAs) were obtained using ClustalW (41) and visualized using the clustal coloring method of Jalview version 2.11.3.2 (42). In addition to *Rp*PKMR1, *Rt*PKMT2 and KmtA, 10 sequences were extracted from each cluster (Cluster A and Cluster R) to create the MSAs.

Table S1. Label-free LC–MS analysis of Tol 5.
Table S2. Peptides with methylation.
Table S3. Peptide fragments from AtaA.
Table S4. Lysine residues in AtaA.
Table S5. Genera found in the two clusters in Figure 3B.
Table S6. Bacterial strains for which the complete genome sequences are available and which have the PKMT genes.

Table S7. Strains and plasmids used in this study.

Strain	Description	Reference
***Acinetobacter* sp.**		
Tol 5	Wild type strain	(48)
REK123Δ*ataA*	Restriction enzyme-encoding genes and *ataA* knockout mutant of Tol 5	(46)
Δ*kmtA*	*kmtA* gene knockout mutant of Tol 5	This study
Δ*ataA*	Unmarked Δ*ataA* mutant of Tol 5	(49)
Δ*ataA*Δ*kmtA*	*kmtA* gene knockout mutant of Δ*ataA* mutant	This study
Escherichia coli		
DH5a	Host for routine cloning	Purchased from Takara (Shiga, Japan)
S17-1	Donor strain for bacterial conjugation	(50)
**Plasmid**		
pBECAb-apr	For gene knockout	(45)
pBECAb-apr-kmtA	For *kmtA* gene knockout	This study
pARP3	*E. coli-Acinetobacter* shuttle expression vector	(14)
pKmtA	Expression vector for KmtA	This study

Table S8. Primers used in this study.

Primer Name	Sequence (5’ to 3’)
kmtA-spacer-BsaI-F	attcgaggtctcatagtTCCAACTCCAAATACCATGCgttttgagaccattgtc
kmtA-spacer-BsaI-R	gtcaatggtctcaaaacGCATGGTATTTGGAGTTGGAactatgagacctcgaat
kmtA-spacerSeq-F	acaacaacgtggaccgatggctac
kmtA-spacerSeq-R	ctttaaagatcggatgctcaatgg
ARP3kmtAF	ccgtttttttgggctagcgcaattataggaaaaaagatgacaacaacg
ARP3kmtAR	cgcggtggcggccgctagtagggatgtctaaatagaagctatgc

References

48. Hori K, Shigeki Y, Shun'ichi I, Masami K, Yasunori T, Hajime U. 2001. Isolation, characterization and application to off-gas treatment of toluene-degrading bacteria. *J Chem Eng Jpn* 34:1120-1126.

49. Ishikawa M, Hori K. 2013. A new simple method for introducing an unmarked mutation into a large gene of non-competent Gram-negative bacteria by FLP/FRT recombination. *BMC Microbiol* 13:86.

50. Simon R, Priefer U, Pühler A. 1983. A broad host range mobilization system for *in vivo* genetic engineering: transposon mutagenesis in gram negative bacteria. *Nat Biotechnol* 1:784-791.
